# Thermally Conductive and Electrically Insulated Silicone Rubber Composites Incorporated with Boron Nitride−Multilayer Graphene Hybrid Nanofiller

**DOI:** 10.3390/nano12142335

**Published:** 2022-07-07

**Authors:** Bangjun Deng, Yangyang Shi, Xiaowen Zhang, Wenshi Ma, Hai Liu, Chunli Gong

**Affiliations:** 1 College of Technology, Hubei Engineering University, Xiaogan 432000, China; sophydeng609@163.com (B.D.); jiayou.xiaowen@163.com (X.Z.); 2 School of Materials Science and Engineering, South China University of Technology, Guangzhou 510640, China; yshi2016@163.com; 3 Hubei Engineering & Technology Research Center for Functional Materials from Biomass, School of Chemistry and Materials Science, Hubei Engineering University, Xiaogan 432000, China; chunli.gong@hbeu.edu.cn

**Keywords:** thermally conductive, electrically insulating, electrostatic self−assembly, boron nitride, multilayer graphene, silicone rubber

## Abstract

Thermally conductive and electrically insulating composites are important for the thermal management of new generation integrated and miniaturized electronic devices. A practical and eco−friendly electrostatic self−assembly method was developed to prepare boron nitride−multilayer graphene (BN−MG) hybrid nanosheets. Then, BN−MG was filled into silicone rubber (SR) to fabricate BN−MG/SR composites. Compared with MG/SR composites with the same filler loadings, BN−MG/SR composites exhibit dramatically enhanced electrical insulation properties while still maintaining excellent thermal conductivity. The BN−MG/SR with 10 wt.% filler loading shows a thermal conductivity of 0.69 W·m^−1^·K^−1^, which is 475% higher than that of SR (0.12 W·m^−1^·K^−1^) and only 9.2% lower than that of MG/SR (0.76 W·m^−1^·K^−1^). More importantly, owing to the electron blocking effect of BN, the electron transport among MG sheets is greatly decreased, thus contributing to the high−volume resistivity of 4 × 10^11^ Ω cm for BN−MG/SR (10 wt.%), which is fourorders higher than that of MG/SR (2 × 10^7^ Ω·cm). The development of BN−MG/SR composites with synergetic properties of high thermal conductivity and satisfactory electrical insulation is supposed to be a promising candidate for practical application in the electronic packaging field.

## 1. Introduction

With the rise of communication technology, artificial intelligence, the Internet, and other technologies, the design of electronic equipment is developing rapidly toward the direction of integration and miniaturization [1]. Due to the significantly increased power density of electronic equipment, efficient heat dissipation leads to urgent demands for thermal interface materials (TIMs) with high thermal conductivity to maintain the life and reliability of electronic devices [2]. Silicone rubber (SR) has been widely used in the field of electronic packaging as TIMs by virtue of its high heat resistance and excellent electrical insulation [3,4]; however, because the average free path of phonons is reduced by the amorphous arrangement of SR molecular chains [5], the thermal conductivity of SR is only about 0.12 W·m^−1^·K^−1^, which cannot meet the requirements of heat dissipation of electronic components under high power density. Adding high intrinsic thermal conductive inorganic fillers such as carbon nanotubes [6,7], SiO_2_ [8,9], and Al_2_O_3_ [10,11] into the SR matrix is the most commonly used method to improve the thermal conductivity of SR.

Graphene is a single−layer carbon atom crystal that isbound together by two−dimensional sp^2^ hybrid bonds. Its unique low dimensional structure can significantly reduce the boundary scattering of phonons at the grain boundary and provide a special phonon diffusion mode in graphene, which endows it with ultra−high thermal conductivity. Balandin et al. [12] found that the thermal conductivity of suspended monolayer graphene was about 5000 W·m^−1^·K^−1^, which is one of the currently known materials with the highest thermal conductivity. It has been proved that the addition of graphene into the SR matrix can efficiently improve the thermal conductivity of the obtained composites. SR/graphene films were fabricated through spin−assisted LBL assembly and the film with 40 assembly cycles has athermal conductivity of 2.03 W·m^−1^·K^−1^ in the in−plane direction [13]. The thermal conductivity of graphene/SR composites with only ~0.7 wt.% of graphene prepared by Tian et al. reaches ~0.3 W·m^−1^·K^−1^ [14,15]. The thermal conductivity of graphene nanoplatelets (GNPs)/SR composites with 8 wt.% of GNPs prepared by Song et al. improves from 0.16 to 0.26 W·m^−1^·K^−1^ [16]; however, there is an inevitable decrease in the electrical insulation property of the graphene−incorporated composite materials due to the high electrical conductivity of graphene, thus resulting in electron leakage or even short circuit of electronic equipment, which is unacceptable in the electronic industry.

In order to solve this problem, attempts have been made to reduce the electrical conductivity of graphene sheets bycoating insulating particles or layers onto the surface of graphene. Many insulating materials (e.g., silica [17,18], alumina [19,20],aluminum [21], and MgO [22]) have been used to block the transport of electrons through the graphene−filled polymer composites. Unfortunately, the heat transfer between graphene and the polymer matrix is hindered to some extent because the thermal conductivities of these coating materials are much lower than that of graphene, thus inevitably sacrificing the thermal conductivity of the composites; therefore, how to fully utilize the excellent thermal conductive ability of graphene while keeping thehigh electrical resistivity of the graphene−incorporated composites is still a challenge. 

Hexagonal boron nitride (BN), a layered insulating material with the same atomic structure asgraphene, possesses similar exotic properties tographite, e.g., relatively high thermal conductivity (~360 W·m^−1^·K^−1^ [23]), mechanical robustness, and thermal stability. It is thus extensively studied for application in the field of thermally conductive and insulating composite materials. The thermal conductivity of SR/BN composite prepared by Ou et al. with 50 wt% BN filler was 0.554 W·m^−1^·K^−1^ [24]. Yin et al. found that BN with an average diameter of 30 μm can efficiently improve the thermal conductivity of SR/BN composites and the in−plane thermal conductivity of the composite reached a maximum value of 6.3 W·m^−1^·K^−1^ [25]. SR incorporated with BN−CNTs hybrid filler exhibited 75% higher thermal conductivity relative to the neat SR [26]. Yang et al. prepared SR/aligned−BN composite sheets by shearing the compound with the two−roll mill; the through−plane thermal conductivity of the composite reached 5.4 W·m^−1^·K^−1^, which was ~33 times higher than that of pure SR [27]. Moreover, two−dimensional BN is also considered to be one of the most promising materials for integration with other 2D materials such as graphene [28] to form new hybrid nanosheets for a wide range of applications; therefore, hybrid fillers prepared by combining boron nitride with graphene is expected to balance the electrical insulating property and thermal conductivity of the obtained silicon rubber/graphene composites. However, due to the high chemical stability or inertness of BN [29,30], it is difficult to hybridize directly with graphene. Surface functionalization of BN is an effective method to promote the combination of the two components. Wei et al. [30] used polydopamine to modify the surface of boron nitride sheets, which was then co−deposited with Ag nanoparticles and mixed into the SR matrix to prepare a thermally conductive as well as electrically insulating composite. The fabricated composite with a filler content of 30 vol.% exhibits a thermal conductivity of 0.75 W·m^−1^·K^−1^ (about 5.76 times that of pure SR) while maintaining a low AC conductivity of 1.89 × 10^−11^ S cm^−1^ at 100 Hz. Xie et al. [31] modified BN by alkylation and then incorporated it with polypropylene matrix to prepare a nanocomposite film with a high thermal conductivity of 2.74 W·m^−1^·K^−1^ and low dielectric loss of only 0.002 (with a low filler loading of 5.5 vol.%). Liu et al. [32] used polyetherimide (PEI) resin to non−covalently modify the surface of boron nitride and composited it with polyetheretherketone (PEEK) matrix. The composite material with 30 wt.% filler loading reaches a high thermal conductivity of 1.01 W·m^−1^·K^−1^. So far, there is little research on the effect of BN/graphene hybrids on the thermal conductivity and electrical insulation of SR composites.

In our previous work, Fe_3_O_4_ functionalized multilayer graphene hybrid was filled into the SR matrix and then induced by an external magnetic field to efficiently enhance the through−plane thermal conductivity to 0.64 W·m^−1^·K^−1^ [33]. A kind of cellulose nanofibers/BN composite film was fabricated and achieved a significant integration of high in−plane thermal conductivity of 15.13 W·m^−1^·K^−1^ [34]. Herein, a further effort to construct a thermally conductive and electrically insulating BN−coated MG hybrid nanosheet by a facile electrostatic self−assembly methodwas carried out in this work. Poly(dimethyldiallylammonium chloride) (PDDA), a strong cationic polyelectrolyte, was selected to functionalize BN nanosheets, considering the fact that Kim et al. [35] and Rouse et al. [36] found PDDA can wrap on carbon nanotubes (CNTs) through the electrostatic interaction between the −COO^−^ of CNTs and the positive charges of PDDA backbone. The positive charges on the backbone of PDDA attract the negative charges on the surface of BN through electrostatic interaction so that PDDA can wrap on the surface of BN to form PDDA functionalized BN (PDDA@BN). Then, the positively charged PDDA@BN can further attract the negative charges on the surface of multilayer graphene (MG) through electrostatic self−assembly to prepare BN coated MG hybrid nanosheets (BN−MG). Since the coating of BN layers onto MG sheets is achieved through a non−covalent bonding way, the integrity of the structure of graphene is not destroyed, which is very important to maintain its high thermal conductivity. BN−MG−filled silicone rubber composites (BN−MG/SR) prepared in this work exhibit excellent electrical insulation performance while maintaining relatively high thermal conductivities.

## 2. Experimental

### 2.1. Materials

Hexagonal boron nitride (BN) with anaverage lateral size of 1 μm was purchased from Suzhou Napu Materials Co., Ltd. (Suzhou, China). Multilayer graphenes (MG) with anaverage lateral size of 1~4 μm were produced by a wet ball milling method reported in our previous work [37]. Poly(dimethyldiallylammonium chloride) (PDDA, 20 wt.% in water) was supplied by Wuxi Lansen Chemicals Co., Ltd. (Wuxi, China).Vinyl silicone oil with vinyl content of 1 wt.% (viscosity = 1000 cps), hydroxy silicone oil containing 0.18 wt.% hydrogen content, and Pt−based catalyst with Pt content of 5000 ppm were all purchased from Dongguan Dongsheng Industrial Co., Ltd. (Dongguan, China). Ethyl acetate (A.R.) and anhydrous ethanol were obtained from Tianjin Damao Chemical Reagent Factory (Tianjin, China).

### 2.2. Preparation of PDDA@BN

In total, 2 g of BN was ultrasonically dispersed in 200 mL of water containing 0.2 wt.% PDDA and 0.5 wt.% NaCl. A homogeneous suspension was formed and then reacted for 12 h at room temperature under continuous stirring. After that, the resulting PDDA functionalized BN (PDDA@BN) was separated by centrifugation, washed, and filtered three times. The final product was dried in a vacuum oven at 70 °C for 12 h.

### 2.3. Preparation of BN−MG

In total, 0.5 g of PDDA@BN and 5 g of MG were ultrasonically dispersed in 500 mL and 1000 mL of water, respectively. Then, the MG dispersion was slowly added to the PDDA@BN dispersion, and the mixed suspension was stirred continuously for 1 h to promote the self−assembly of PDDA@BN and MG through the electrostatic interaction between the two. After, the final boron nitride−multilayer graphene hybrid nanosheets (BN−MG) with a 1:10 mass ratio of BN to MG were obtained by precipitation, filtration, and vacuum−drying at 70 °C for 12 h.

### 2.4. Preparation of BN−MG/SR Composites

The BN−MG/SR composites with different loading of BN−MG (1 wt.%, 3 wt.%, 5 wt.%, 7 wt.%, and 10 wt.%) were prepared by the following solution blending method: Firstly, a certain amount of BN−MG was ultrasonically dispersed into ethyl acetate, and then vinyl silicone oil and hydroxy silicone oil were added into the BN−MG dispersion and stirred uniformly. Subsequently, Pt catalyst was added to the above mixture and stirred uniformly. After solvent volatilization in a fume hood, the mixture was vacuum pumped to remove the air bubbles. Finally, the de−bubbled mixture was poured into a polypropene (PP) mold and heated to 110 °C for 5 h to obtain fully cured BN−MG/SR composites. For comparison purposes, multilayer graphene−filled silicone rubber composites (MG/SR) with the same filler loadings were also prepared. 

### 2.5. Characterization

The Zeta potentials of MG, BN, and PDDA@BN were measured with a nano−laser particle size analyzer (Zatasizer Nano−ZS, Malvern). Before measurement, the samples were uniformly dispersed in water. The crystal structure of MG, PDDA@BN, and BN−MG was examined on an X−ray diffractometer (XRD, X’Pert Pro, PANalytical) with Cu Kα radiation (*λ* = 0.154 nm) at a step size of 0.06° in the 2θ range of 10°~80°. The chemical structure of MG, BN, PDDA@BN, and BN−MG was characterized using a Fourier transform infrared spectrometer (FT−IR, IRTracer−100, SHIMADZU) with a scanning range of 4000~400 cm^−1^. The thermal stabilities of BN, PDDA@BN, MG, BN−MG, MG/SR, and BN−MG/SR samples were analyzed on a thermogravimetric analyzer (TGA55, TA) at a heating rate of 20 °C·min^−1^ in a nitrogen flow (20 mL·min^−1^) from 40 °C to 800 °C. The thermal conductivity and thermal resistance of BN−MG/SR and MG/SR samples were tested by a heat flow method on an automatic thermal conductivity tester (DRL−III, Xiangtan Xiangyi Instrument Co., Ltd., Xiangtan, China).The samples with a thickness of 1~3 mm were cut into a circle with a diameter of 3 cm, and the applied pressure was 100 N. The volume resistivity of MG/SR and BN−MG/SR samples with a diameter of 10 cm and a thickness of 1~3 mm were tested by an electrometer (KEI−6517A, Keithey) according to Chinese Standard (GB/T1410−2006). The threeelectrodes method was used to measure the volume resistance (Rx) of the sample, and the volume resistivity of the sample was obtained after calculation according to Formula (1).For the test of thermal conductivity and volume resistivity, at least three duplicate samples were tested, and the average value was taken.The morphology of BN, MG, BN−MG, BN−MG/SR, and MG/SR was observed by scanning electron microscope (SEM, S−3700N, Hitachi). The powder samples (BN, MG, and BN−MG) were dispersed in ethanol and dropped onto a silicon sheet. The fresh cross−sections of BN−MG/SR and MG/SR composite samples were obtained by tensile fracture.

(1) $$\mathrm{VR}=\mathrm{Rx}\;\times \frac{A}{h}$$

where VR is the volume resistivity of the sample (Ω cm), Rx is themeasured volume resistance of the sample (Ω), A is the effective area of the protected electrode (cm^2^), and h is the average thickness of the sample (cm).

## 3. Results and Discussions

### 3.1. Characterization of BN−MG

**Figure 1:**
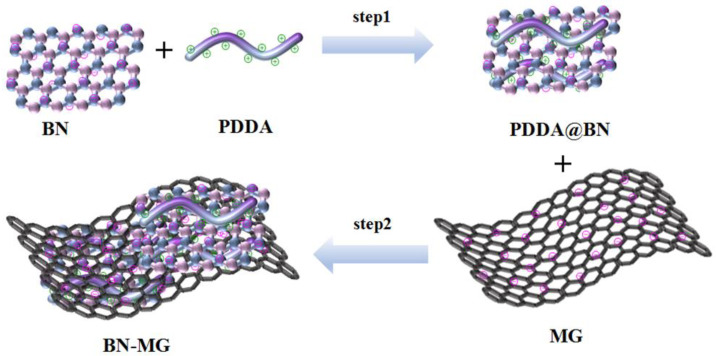
The schematic of the preparation route of BN−MG.

The schematic of the preparation route of the BN−MG hybrid nanosheets is shown in Figure 1. Since there are multiple cations on the backbone of PDDA polyelectrolyte, the addition of NaCl during “step 1” can partly shield the charges of PDDA, thus promoting the molecular chains of PDDA to adopt random conformation [38,39]. Compared with the rigid rod conformation of PDDA formed without NaCl addition, the attraction between the positive charges on the backbone of PDDA with random conformation and the negative charges on the surface of BN is increased, and therefore, more PDDA can wrap on the BN surface to form PDDA−functionalized BN (PDDA@BN). In the following “step 2”, the positively charged PDDA@BN can further attract the negative charges on the surface of graphene and lead to the coating of BN layers on the surface of graphene sheets through electrostatic self−assembly. 

**Figure 2:**
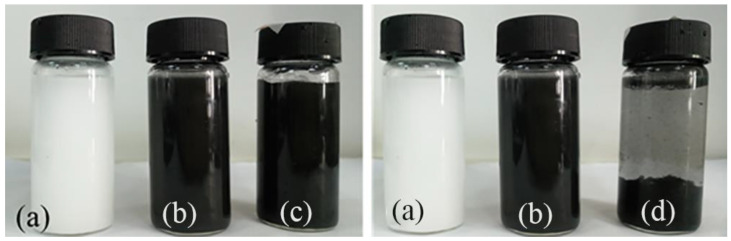
Photos of PDDA@BN/water dispersion (**a**), MG/water dispersion (**b**), the mixture dispersion of PDDA@BN and MG just after mixing (**c**) and after 30 min (**d**) as a result of electrostatic self−assembly.

The photographs of the samples during the process of electrostatic self−assembly of BN−MG are shown in Figure 2. In order to realize the coating of BN on the surface of MG sheets, the cationic polyelectrolyte PDDA was used to functionalize BN first. As shown in Figure 2a, PDDA@BN could be uniformly dispersed in water. When the PDDA@BN water dispersion was mixed with MG dispersion with the mass ratio of 1:10 (PDDA@BN:MG), the positively charged PDDA@BN combined with the negatively charged MG through electrostatic self−assembly and the mixture gradually settled down in the aqueous dispersion after 30 min, as shown in Figure 2c,d. This electrostatic self−assembly process is practical and eco−friendly, which is suitable for large−scale preparation.

**Figure 3:**
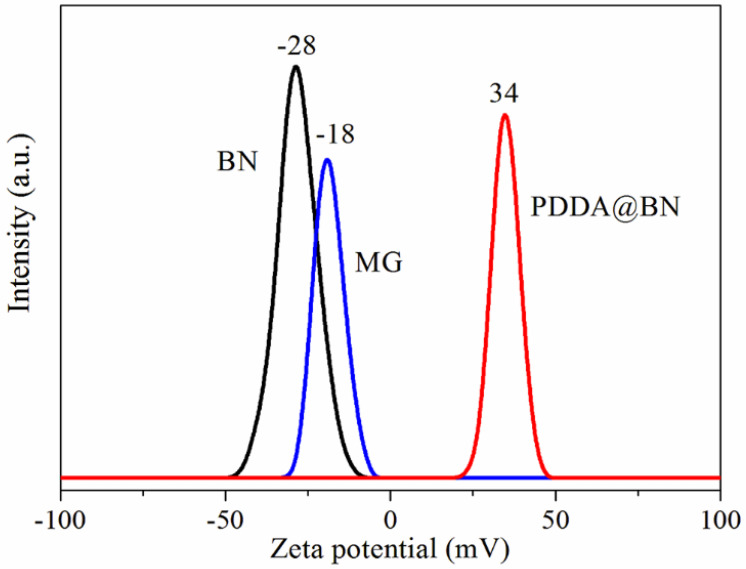
Zeta potential curves of BN, MG, and PDDA@BN.

Zeta potential can directly reflect the change of charges in the dispersions; the obtained Zeta potential curves are shown in Figure 3. As for BN and MG, the Zeta potentials of the two samples are −28 mV and −18 mV, confirming that both BN and MG are negatively charged, which is due to the oxygen−containing functional groups such as hydroxyl on the surface of BN and MG. After functionalization by PDDA, the zeta potential of BN changes from −28 mV to 34 mV, which verifies that PDDA with multiple positive charges successfully wrapped on the surface of BN, making the surface of BN positively charged. The increase in the absolute value of Zeta potential indicates that BN can be dispersed more uniformly and stably in an aqueous solution after functionalization by PDDA.

**Figure 4:**
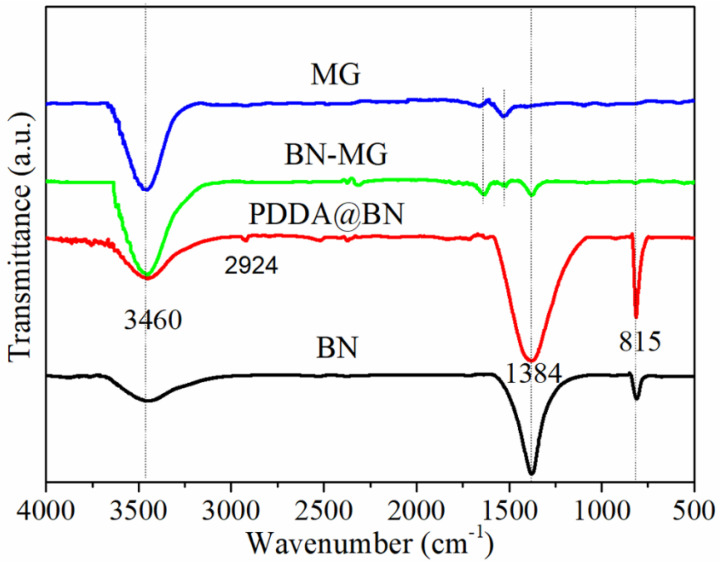
FT−IR spectra of BN, PDDA@BN, MG, and BN−MG.

FT−IR spectra of BN, PDDA@BN, MG, and BN−MG are shown in Figure 4. As for BN, the two strong absorption peaks at 1384 cm^−1^ and 815 cm^−1^ correspond to the in−plane stretching vibration and out−of−plane bending vibration of the B−N bond, respectively. Moreover, there is another strong absorption peak at 3460 cm^−1^, which is attributed to the stretching vibration of the hydroxyl group on the surface of BN. When compared with the FTIR spectrum of BN, the functionalized PDDA@BN exhibit the stretching vibration of the C−H bond at 2924 cm^−1^, indicating that PDDA has already been introduced into BN [38,39]. From the FTIR spectrum of MG, two main peaks at 3460 cm^−1^ and 1640 cm^−1^ are attributed to the stretching vibration of hydroxyl groups and stretching vibration of C=C bonds of MG, respectively. As expected, the characteristic absorption peaks attributed to BN and MG can be clearly observed on the FTIR spectrum of BN−MG, which verifies the successful preparation of BN−MG.

**Figure 5:**
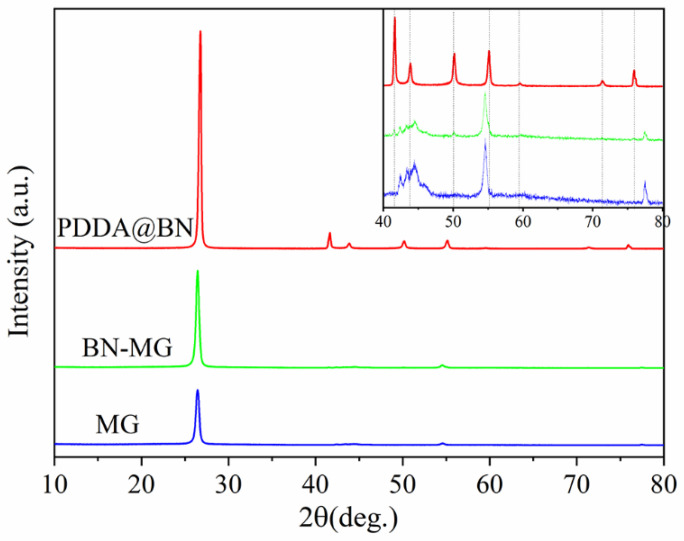
XRD patterns of PDDA@BN, MG, and BN−MG.

The crystal structure of PDDA@BN, MG, and BN−MG are shown in Figure 5. The strong and sharp peak at 2θ = 26.0° is the characteristic peak of MGcorresponding to the (002) crystal plane diffraction of MG. The strong characteristic (002) crystal plane diffraction peak of BN is at 2θ = 26.9°. Diffraction peaks at 2θ =41.6°, 43.8°, 50.2°, 55.1°, and 76.0° correspond to the (100), (101), (102), (004), and (110) crystal plane of BN in accordance with the reference data of JCPDS card No.34−0421. When compared with MG, BN−MG has five new diffraction peaks at 2θ = 41.6°, 43.8°, 50.2°, 55.1°, and 76.0°. These new five diffraction peaks correspond to the (100), (101), (102), (004), and (110) crystal planes of BN and further confirm that BN had successfully coated on the surface of MG through electrostatic self−assembly.

**Figure 6:**
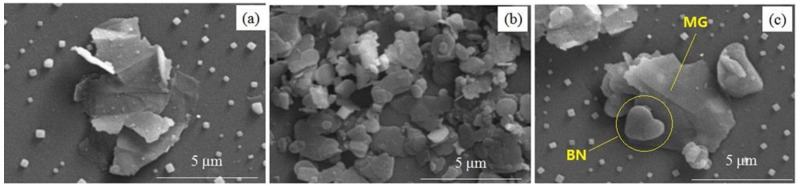
SEM images of MG (**a**), BN (**b**), and BN−MG (**c**).

The micro−morphology of BN, MG, and BN−MG are shown in Figure 6. The lateral size of MG sheets is 1~4 μm, which can be confirmed by the SEM image of MG in Figure 6a. It can be seen from Figure 6b that the lateral size of BN is about 1 μm. BN is sheet−like with a smooth surface and a relatively uniform size distribution. The size difference between BN and MG makes it possible for BN to coat the surface of MG. As shown in Figure 6c, BN sheets stably coat the surface of MG sheets. This coating can reduce the direct contact between MG sheets, thereby reducing the electrical conductivity of graphene. Since the mass ratio of BN to MG in the BN−MG hybrid nanosheet is only 1:10, as well as the density of BN, is much larger than that of MG, BN with fewer lamellae cannot completely coat the surface of MG, which is of great significance for maintaining the high thermal conductivity of BN−MG. 

**Figure 7:**
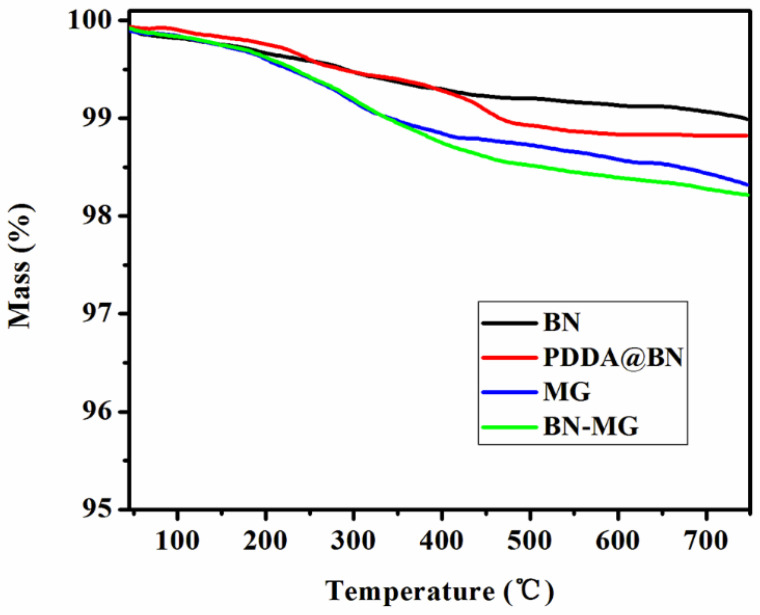
TGA curves for BN, PDDA@BN, MG, and BN−MG.

TGA tests were carried out under a nitrogen atmosphere to evaluate the thermal stabilities of BN, PDDA@BN, MG, and BN−MG. The obtained curves are shown in Figure 7. BN shows negligible weight loss (less than 1% at 750 °C), demonstrating its high thermal stability. As for PDDA@BN, two degradation stages before 450 °C can be found in its TGA curve. The first stage in the range of 100~200 °C is the volatilization of small molecular impurities such as trace moisture remaining in the sample. The second stage, between 300 °C and 400 °C, is mainly due to the decomposition of PDDA. Because of the small wrapping amount of PDDA in PDDA@BN, no further weight loss above 450 °C can be found after the decomposition of PDDA and thus maintains good thermal stability. By comparison, the thermal stability of MG sheets prepared by the wet ball milling method is not as good as that of BN due to the presence of hydroxyl groups on the MG surface; however, the weight loss value of MG and BN−MG at 750 °C is only 1.7 wt.% and 1.8 wt.%, respectively, proving the good thermal stability of both MG−based nanofillers. 

### 3.2. Characterization of BN−MG/SR

**Figure 8:**
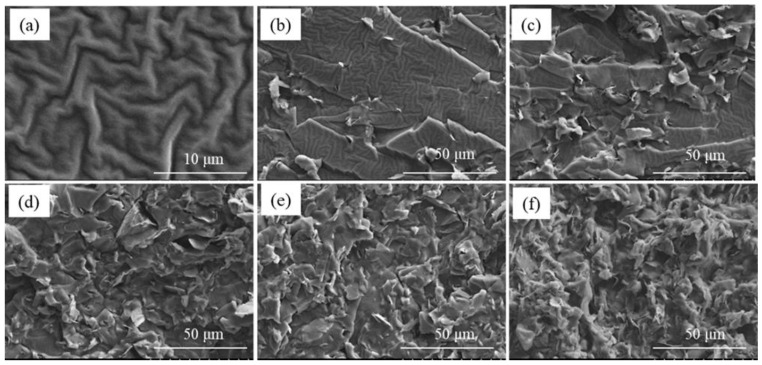
SEM images of BN−MG/SR with different BN−MG loadings: (**a**) 0 wt.%, (**b**) 1 wt.%, (**c**) 3 wt.%, (**d**) 5 wt.%, (**e**) 7 wt.%, and (**f**) 10 wt.%.

The suitable dispersion and compatibility of inorganic nanofillers in polymer−based composites are crucial for the determination of the performance (e.g., mechanical properties, thermal conductivity) of the composites. The cross−sectional images of the BN−MG/SR composites with different BN−MG loadings are shown in Figure 8. It can be seen from Figure 8a that the pure silicone rubber shows a relatively smooth morphology with many wave−like folds. As for the composites (Figure 8b–f), the BN−MG hybrid fillers are uniformly dispersed in silicone rubber matrix without obvious agglomeration, indicating that the self−agglomeration tendency of MG has been reduced after BN coating. In addition, with the increase in BN−MG loading, the cross−sections of the composites become rougher and rougher, and the boundary between fillers and silicone rubber matrix becomes blurred. Such change can further increase the contact areas between the BN−MG hybrid fillers and polymer matrix, which can promote the formationof a continuous thermal conductive network in the composites.

**Figure 9:**
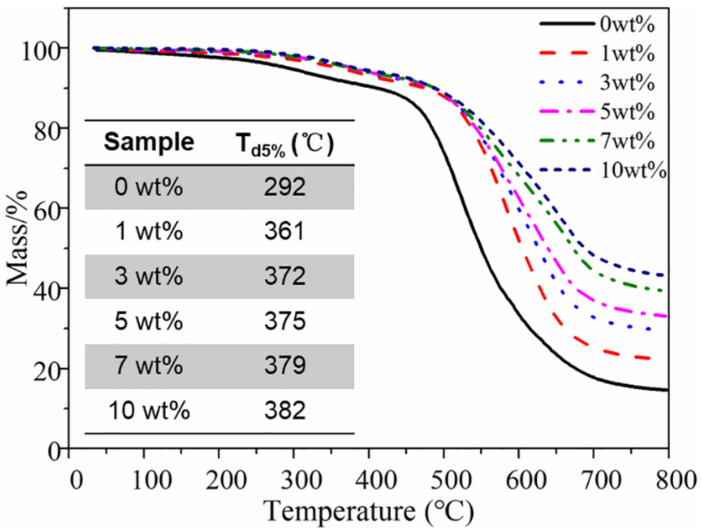
TGA curves of BN−MG/SR with different BN−MG loading.

Thermal stability is one of the most important properties of composite materials, which affects the processability and service life of composites. Figure 9 shows the TGA curves of pure SR and BN−MG/SR composites with different filler loadings. It can be seen that the pure silicone rubber starts to decompose at 292 °C and the main decomposition stage is between 400 °C and 600 °C. The thermal degradation curves of BN−MG/SR composites are similar to that of pure SR, indicating that the addition of BN−MG does not change the degradation mechanism of the silicone rubber matrix. By comparing the initial decomposition temperature (T_d5%_, temperature at 5% weight loss), as shown in the inner table of Figure 9, the thermal stabilities of BN−MG/SR composites are increased significantly when compared with that of pure SR. For example, the T_d5%_ of pure SR is only 292 °C, whereasthetemperature for BN−MG/SR with 10 wt.% filler loading is as high as 382 °C, which is an increase of about 31%. The increase is related to the high thermal stability of the inorganic nanofiller BN−MG (as revealed in Figure 7). Moreover, the homogeneous dispersion of BN−MG in the composites can also play the role of physical crosslink points, which therefore partly prevent the polymer chains from moving, thereby improving the resistance of the silicone rubber matrix to thermal degradation. 

**Figure 10:**
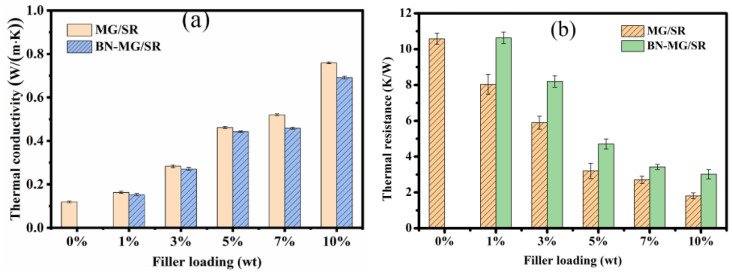
Thermal conductivity (**a**) and thermal resistance (**b**) of MG/SR and BN−MG/SR with different filler loading.

BN−MG/SR and MG/SR with different filler loadings were prepared to compare the effect of functionalized nanofillers on the thermal conductivity and thermal resistance of the composites. As shown in Figure 10a,b, it can be seen that with the increase inthe nanofiller loading, the thermal conductivities of BN−MG/SR and MG/SR composites are increased, while the thermal resistance values of both show a downward trend. For the pure SR, the thermal conductivity is only 0.12 W·m^−1^·K^−1^ and its thermal resistance is nearly 10.6 K·W^−1^. The introduction of MG and BN−MG significantly improves the heat conduction ability of the composites. As for the BN−MG/SR composite with 10 wt.% nanofiller loading, it exhibits a very high thermal conductivity of 0.69 W·m^−1^·K^−1^ as well as a low thermal resistance of 3.0 K·W^−1^, which is nearly 475% higher and 72% less than the values of the pure SR, respectively. In addition, we find that the thermal conductivities of the BN−MG/SR composites are slightly lower than those of the MG/SR composites with the same nanofiller loadings (e.g., 0.69 W·m^−1^·K^−1^ for BN−MG/SR−10% vs. 0.76 W·m^−1^·K^−1^ for MG/SR−10%). It is well known that there is a big difference inthermal conductivity between BN (~360 W·m^−1^·K^−1^ [23]) and MG (~5000 W·m^−1^·K^−1^ [12]).The reason why the BN−MG/SR composites still maintained high enough thermal conduction ability mightbe related to the following reasons: (i) the reduced self−agglomeration tendency of MG after coating by BN and good dispersion of BN−MG fillers in the silicone rubber matrix. As shownin Figure 8, with the increase in BN−MG loadings, the boundary between fillers and SR matrix becomesblurred;thus, more thermal conductive pathways formed, and the continuity of thermal conductive networks increased; (ii) the functional groups could contribute to forming greater interfacial adhesion between BN−MG fillers and SR matrix by covalent bonding or van der Waals’ force, which helps to decrease the interfacial resistance and generate more thermal conductive pathways and networks [40,41]; (iii) BN’s partly coating on MG sheets is of great significance for maintaining the high thermal conductivity of BN−MG. 

**Figure 11:**
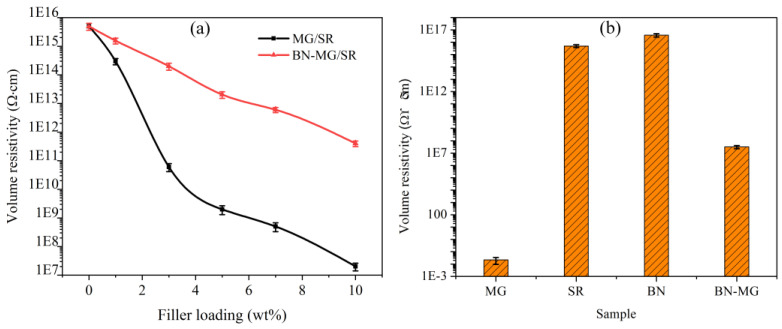
Volume resistivity of MG/SR and BN−MG/SR with different filler loadings (**a**) and volume resistivity of the fillers (**b**).

In addition to the thermal conduction ability, the composites should also possess high electrical resistivity in order to meet the insulation requirement of electronic packaging materials. Figure 11a shows the volume resistivity of the BN−MG/SR and MG/SR composites with different filler loadings. The volume resistivity of both BN−MG/SR and MG/SR shows a decreasing trend when compared with that of pure SR. As shown in Figure 11b, the volume resistivity of MG and BN−MG hybrid is about 10^−2^ Ω·cm and 10^7^ Ω·cm, respectively. So, the filling ofMG or BN−MG will lead to adecrease inelectrical resistivity of the SR matrix. The volume resistivities of the BN−MG/SR composites are significantly higher than those of MG/SR with the same filler loading, showing their relatively high electrical insulating property. As for the MG/SR composites, due to the super high electrical conductivity of MG with graphite structure, the volume resistivities of the composites sharply decrease from 5 × 10^15^ Ω·cm to only 2 × 10^7^ Ω·cm, which drops off by eightorders of magnitude and is far below the critical resistivity for electrical insulation (1.0 × 10^9^ Ω·cm [42]). By contrast, the BN−MG/SR composite with 10 wt.% loading of BN−MG still maintains a high resistivity of 4 × 10^11^ Ω·cm, which decreases by only fourorders of magnitude and satisfies the requirements of electrical insulation. 

**Figure 12:**
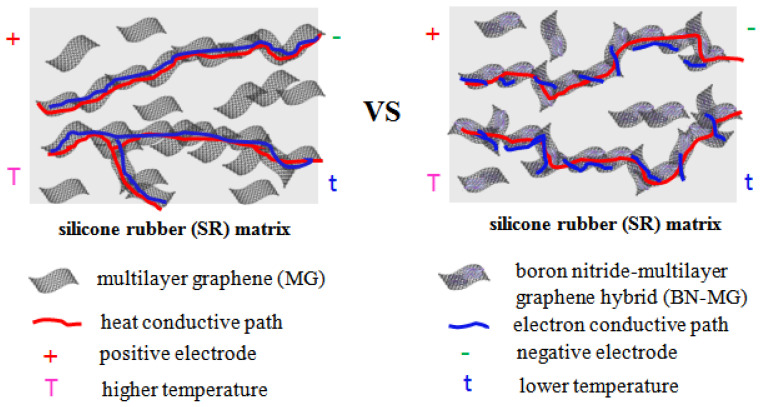
Heat and electron conductive path schematic of SR/MG and SR/BN−MG composites.

Takingthe thermal conductivity (as shown in Figure 10) and insulating property (as shown in Figure 11) into account, the BN−MG/SR with 10 wt.% BN−MG loading exhibits both good thermal conductivity and electrical insulation properties. The thermal conductivity BN−MG/SR with 10 wt.% filler loading is 0.69 W·m^−1^·K^−1^, which is only 9.2% lower than that of MG/SR with the same filler loading. In contrast, the volume resistivity of the former is maintained at 4 × 10^11^ Ω·cm, which is fourorders of magnitude higher than that of the latter. The reason why BN−MG/SR composites show synergetic properties of high thermal conductivity and satisfactory electrical insulation can be explained by the schematic shown in Figure 12. Continuous heat and electron conductive paths can be established in the SR matrix by direct contact among MG sheets, but the direct contact among MG sheets is effectively hindered by the BN layers coating the surface of MG. BN is thermally conductive but also insulating, thereby, the conductive heat paths in the SR matrix are still maintained, but the electron conductive paths are greatly blocked. In addition, we also compared the thermal conductivity and volume resistivity of the BN−MG/SR composite (with 10 wt.% filler loading) with those of other SR−based composites reported in references (as listed in Table 1). The result proved that BN−MG hybrid filler prepared in this study via the practical and eco−friendly electrostatic self−assembly method is a promising candidate as a filler for practical application in the field of electronic packaging.

## 4. Conclusions

A practical and environmentally friendly electrostatic self−assembly method was used to successfully prepare BN−MG hybrid nanosheets. The obtained BN−MG was then added as a novel thermally conductive and insulating filler into the silicone rubber matrix and cured to prepare BN−MG/SR composites. In the case of filler loading below 10 wt.%, the BN−MG/SR composites exhibit a comparable thermal conductivity to that of MG/SR with the same filler loading. The volume resistivity of BN−MG/SR is much higher than that of MG/SR due to the electron transport between MG sheets being blocked after BN lamellae partially coatedthe surface of MG sheets. The thermal conductivity of the BN−MG/SR composite (10 wt.% BN−MG loading) is 0.69 W·m^−1^·K^−1^, which is only 9.2% lower than that of MG/SR with the same filler loading, while its volume resistivity is fourorders of magnitude higher than that of the latter. 

## Figures and Tables

**Table 1:** Comparison of thermal conductivity (TC) and volume resistivity (VR) of the BN−MG/SR composite in this work with those of other SR−based composites.

Filler	Filler Loading	TC (W·m^−1^·K^−1^)/I *	VR (Ω·cm)	References/Year
Graphene (G)	3 wt.%	−	2 × 10^5^	[43] 2016
G	1.8 wt.%	−	3.57 × 10^5^	[44] 2022
G	2.53 wt.%	2.03/786%	−	[13] 2018
G	0.72 wt.%	0.30/50%	−	[15] 2017
G	0.67 wt.%	0.305/45%	−	[14] 2017
Boron nitride (BN)	50 phr	0.25/56%	2.5 × 10^16^	[27] 2019
BN	28 vol.%	0.4/122%	−	[25] 2021
BN	12.59 vol.%	0.225/40.6%	3.5 × 10^16^	[26] 2018
BN	20 wt.%	0.24/50%	3 × 10^16^	[24] 2019
Al_2_O_3_−G hybrid	31 wt.%	−	5.1 × 10^12^	[20] 2021
Silica−G hybrid	2 wt.%	0.497/155%	9.23 × 10^12^	[18] 2018
BN−G hybrid	10 wt.%	0.69/475%	4 × 10^11^	This work

*: I is the TC increment of composites listed in Table 1 compared to the referential SR sample for each work.
